# Clustering and graph mining techniques for classification of complex structural variations in cancer genomes

**DOI:** 10.1038/s41598-022-07211-6

**Published:** 2022-02-28

**Authors:** Gonzalo Gomez-Sanchez, Luisa Delgado-Serrano, David Carrera, David Torrents, Josep Ll. Berral

**Affiliations:** 1 Department of Computer Science, Barcelona Supercomputing Center (BSC), 08034 Barcelona, Spain; 2 Universitat Politècnica de Catalunya (UPC), 08034 Barcelona, Spain; 3 Department of Life Science, Barcelona Supercomputing Center (BSC), 08034 Barcelona, Spain; 4 Institució Catalana de Recerca i Estudis Avançats (ICREA), 08010 Barcelona, Spain

**Keywords:** Data mining, Data processing, Functional clustering, Statistical methods, Cancer genomics, Genomics

## Abstract

For many years, a major question in cancer genomics has been the identification of those variations that can have a functional role in cancer, and distinguish from the majority of genomic changes that have no functional consequences. This is particularly challenging when considering complex chromosomal rearrangements, often composed of multiple DNA breaks, resulting in difficulties in classifying and interpreting them functionally. Despite recent efforts towards classifying structural variants (SVs), more robust statistical frames are needed to better classify these variants and isolate those that derive from specific molecular mechanisms. We present a new statistical approach to analyze SVs patterns from 2392 tumor samples from the Pan-Cancer Analysis of Whole Genomes (PCAWG) Consortium and identify significant recurrence, which can inform relevant mechanisms involved in the biology of tumors. The method is based on recursive KDE clustering of 152,926 SVs, randomization methods, graph mining techniques and statistical measures. The proposed methodology was able not only to identify complex patterns across different cancer types but also to prove them as not random occurrences. Furthermore, a new class of pattern that was not previously described has been identified.

## Introduction

Cancer is a complex disease that is normally triggered by changes (mutations) in the genome of a given cell. Although some cancer types are promoted by germline variants (i.e. those that we inherit from our parents), the vast majority of them are caused by somatic changes in the genome that occur during our life and are not passed onto the offspring. These somatic changes are triggered by internal cellular processes, as well as by several environmental and life-style factors, such as smoking, or nutrition, among others. Understanding which are the variants responsible for the development and progression of tumors is key to understanding and designing clinical protocols for the prediction or treatment of this complex disease.

For the last few years, several large initiatives have been gathering and analyzing genomic sequences of thousands of different tumors (see below). From these analyses, we now know that there are different types of somatic variants playing a role in the biology of the tumor, covering from single substitutions, to large chromosomal rearrangements. A particularly important class of somatic alterations related to cancer are the structural variants (SVs) that consist of the modification of large portions of the genome, in the form of large chromosomal rearrangements, which can include deletions, insertions, tandem duplications, inversions, and translocations^1^. Furthermore, we have also learned that an important fraction of SVs are not independent and random events but are acquired through a “single-hit” event involving several DNA breaks, usually resulting in complex genome rearrangements, which are normally correlated with the aggressivity of the tumor. Although it is key to understand the mechanisms behind these complex events, there is currently not a standard methodology to identify and classify such events, and only a few cases have been so far described.

In 2011, Stephens and co-workers described an SV pattern characterized by multiple (sometimes hundreds) rearrangements that occur within a restricted portion of the genome, involving normally one, but also rarely two chromosomes^2^. In another study, Baca et al. reported another specific pattern of chromosomal rearrangements in prostate tumors called Chromoplexy, which is characterized by a closed chain of translocations involving several chromosomes^3^. Recently, the PCAWG Consortium collected whole genome sequencing data from 2392 tumors across 36 cancer types, produced by the International Cancer Genome Consortium (ICGC) and The Cancer Genome Atlas (TCGA) projects^4^. There, Li and co-workers described a replication-based mechanism of structural variation that results in varied chromosomal structures with low-level copy number gains and recurring inverted rearrangements^5^. Despite all these efforts to classify and characterize these complex events, a major fraction of the identified SVs in the PCAWG study remained “unclassified.”

In order to fulfill this gap, we developed an innovative statistical approach to be able to discriminate between stochastic chromosomal rearrangements, probably due to general genome instability^6^, from those patterns that might have specific and recurrent molecular mechanisms behind them. The generation of such a workflow will allow the overall improvement of classification methods for the discrimination of mutations and to identify particular SV signatures as markers of tumor formation and progression.

Here, we applied this new statistical frame to 2392 tumor genomes from the PCAWG Consortium, including more than 152,926 SVs. These tumor genome samples are classified into 36 different cancer types (sample distribution can be found in Table S1), each of them containing the information of their particular SVs. The SVs are encoded in Variant Call Format files (VCF files), where each SV is described as a novel adjacency of two breakends. These breakends appear when a chromosome is broken at a given locus (breakpoint). The adjacency refers to the SV junction that ties together two breakends. A schematic representation can be found in Fig. S1.

The method developed takes into account the local distribution of SVs in every sample and is optimized using the global distribution across the dataset, using a Kernel Density Estimation function^7,8^. The aim of the clustering is to join the rearrangements that are likely derived from the same molecular mechanism, as they share some topological properties. We assessed that the clustering approach joins rearrangements not randomly by performing a permutation test. Then, we provided a graph mining method to analyze the SV patterns, using advanced high-performing technologies to reduce the computational cost^9,10^. Finally, we adapted a methodology proposed by Wong^11^ to obtain the level of significance of the different patterns based on the Abundance, a measure that indicates the overrepresentation or underrepresentation of a pattern against a random scenario.

By overcoming currently unsolved challenges of SVs classification in cancer, our results provide insights towards the better identification of tumor progression markers that can be used to predict and prevent potential situations of bad prognosis.

## Methods

**Figure 1:**
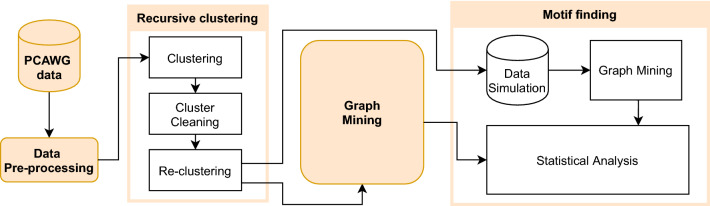
Workflow applied to identify complex rearrangements in PCAWG genomes. Simple data pre-processing was performed before implementing the recursive clustering. Then, the graph mining method was applied to find patterns. Finally, the motif finding strategy was applied to determine the statistically significant patterns.

Our main strategy for the identification of complex chromosomal rearrangements is summarized in Fig. 1. Preceded by a quality check and pre-processing of the PCAWG data, the main workflow is composed of three major steps: KDE clustering, graph mining, and motif finding.

### Defining clusters to identify the SVs involved in complex rearrangements

The clustering method was developed following a mode hunting approach, a modal clustering strategy^12^ where every SV is assigned to a cluster. It has been based on the Kernel Density Estimation (KDE)^7,8^, a non-parametric statistical method to estimate the probability density function of a random variable.

**Figure 2:**
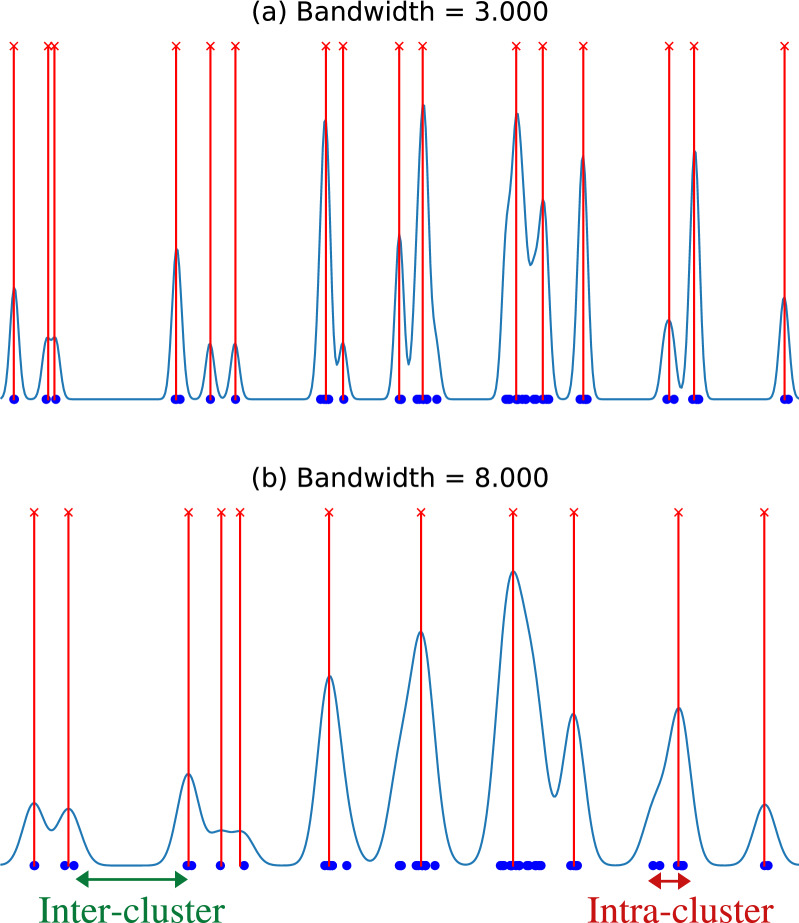
Kernel Density Estimation of breakpoint clusters from chromosome 3 setting bandwidth values of (**a**) 3000 and (**b**) 8000. Blue dots represent the locations of the breakpoints, the blue line is the kernel density estimation and red lines the obtained cluster peaks. The inter and intra-cluster distances are shown in green and red, respectively.

In this study, the random variable is the position of the SV, which is defined by the breakpoints. Clustering those breakpoints that correspond to the same single rearrangement event is crucial to later classify complex patterns of SVs. We chose this clustering method because it uses a density estimation of the breakpoints as a starting point, which allowed us to rely both on the closeness of the breakpoints and their density. Using the Gaussian Kernel based on normal distribution, the only hyperparameter to be set was the before mention bandwidth^13,14^. This value defines how the density estimation is going to be: increasing the bandwidth leads to larger (and fewer) clusters, whereas low values generate smaller and sparser clusters. The final size of each cluster will depend on both the selected bandwidth and the density of the breakpoints for each particular case. Our interest is to find clusters small enough to contain breakpoints from only a rearrangement event while they are far apart from each other, meaning they are two different events. Having this objective, instead of using a strategy to find the optimal bandwidth based on the density (an approach that could also be a valid option, see^15–17^), we decided to set a bandwidth that provides the lowest intra-cluster distance, defined as the highest distance between two breakpoints within the same cluster, and the highest inter-cluster distance, defined as the lowest distance between two breakpoints of adjacent clusters, both illustrated in Fig. 2. These distances were obtained for all samples at the same time, fixing the same bandwidth value for every sample. Therefore, taking into account the global breakpoints distribution across all the samples to set the bandwidth value, we were able to avoid potential biases derived from a particular sample distribution and to join together two clusters or not when needed.

Since the human genome is organized into 23 pairs of chromosomes, we performed the clustering locally at every chromosome. Figure 2 shows how the method works using different bandwidth values on the same region of a given chromosome. Once the clustering was done, the next step was to locate all the peaks of the function and assign the breakpoints to the closest peak. These peaks represent the cluster centers to use for all the breakpoints assigned to each cluster at the graph mining step (see below).

In order to improve the clustering resolution, a recursive 2-step clustering was carried out: after the first KDE clustering process, we performed a second clustering inside every cluster. To avoid already described complex patterns, such as Chromothripsis, the breakpoints looping over the same region were discarded after the first clustering round. This process made the mining of motifs computationally more efficient, avoiding noise into the second step of the clustering. In the second round of clustering, different bandwidth hyper-parameters were set to compare intra-cluster and inter-cluster distances. Notice that after the second round of clustering, original clusters are discarded and only the new obtained clusters remain, providing different values of intra-cluster and inter-cluster distances inside every chromosome. Since the clustering method was based on a density estimation function, we ensured a linear growth of the number of operations with the increase of data. Since both the density estimation and the final cluster selection only interact with data from a region of the chromosome at a time, the number of operations of the method will always be smaller than $\hbox {n}^{2}$, where n is the number of breakpoints, avoiding high computational expenses. To provide a better understanding of the method, the pseudocode of the full clustering process can be found in Algorithm 1.

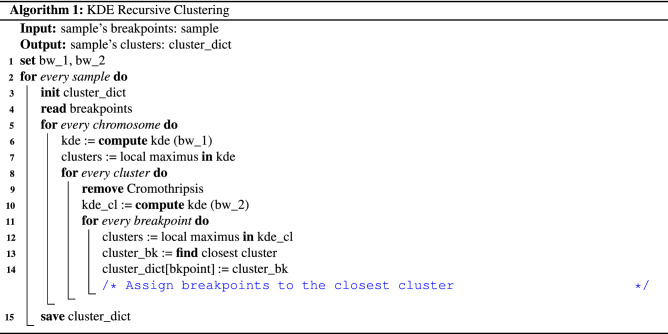



We validated that our clustering approach was not joining random SVs by performing two tests. First, we generated simulated datasets 100 times by pooling together all the breakpoints of the samples, and creating new samples with random rearrangements. We used this method over total randomization to keep the original locations of the SVs since it has been proven that they tend to occur in the same areas of the genome^18^. With these simulated datasets we are trying to determine if the chromosomal rearrangements are independent rearrangements that simply tend to happen in the same places or are dependent, meaning that they tend to occur close by. Past studies about the whole-genome analysis^6^ indicate that they should have some grade of dependency.

In the first test, we estimated the average dispersion of breakpoints in each simulated dataset. We used as a dispersion measure the standard deviation of the difference of base pair between adjacent breakpoints in a chromosome. Then, we compared the average dispersion distribution from the simulated datasets against the average dispersion of breakpoints in the original dataset performing a one sample Z-Test. In the second test, we applied the KDE clustering method to each simulated dataset as described for the original dataset. For each permutation we calculated the average cluster density defined as the average number of breakpoints per cluster and compared to the average number of breakpoints per cluster in the original dataset using a one sample Z-Test. Despite the obtained clusters are based on the KDE, since our objective is to evaluate the similitude between the clusters and not the density function itself, we decided to implement this method over other strategies that focus directly on the comparison between the density functions^19^.

### Graph mining to search for complex rearrangements

**Figure 3:**
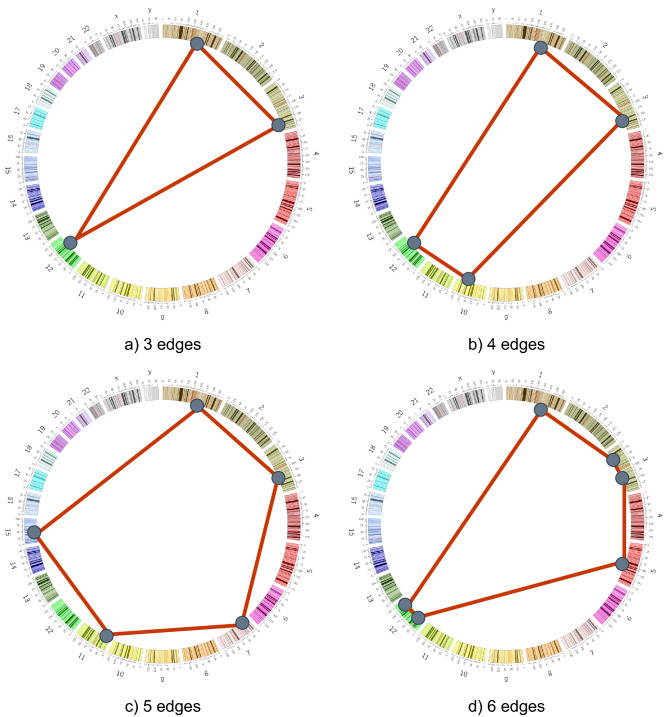
Circular representation of human genome with *cycles* of different sizes .

The clustering process set out every sample as a graph where the breakpoint clusters are represented as vertices and the edges connecting these vertices correspond to the rearrangements. Since vertices could be composed of several breakpoints from different rearrangements, different graphs could be generated. To narrow down the survey of graphs, we focused only on Hamiltonian cycles (mentioned further only as *cycles*), where every vertex is connected to two other vertices (Fig. 3).

To find and count rearrangement patterns inside each graph, we used a search approach method based on the VSIGRAM method^20^, following a vertical approach and finding the frequent subgraphs in a depth-first fashion. As the subgraph mining problem becomes computationally hard (NP-hard), we performed a pruned search with max size = 6. The graph-based data mining for SV pattern searching includes four steps: deduplicate edges, generate the graph, subgraph mining, and reduce similar patterns.

#### Deduplicate edges

Since every cluster can include more than one breakpoint, it is likely to find clusters with more than one edge going to one another cluster. These edges were therefore duplicated and had to be deduplicated, simply removing all of them except one.

**Figure 4:**
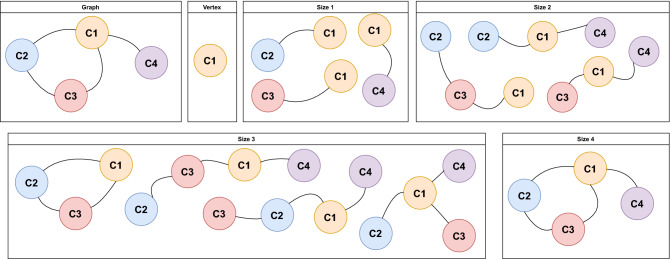
Graphic representation of the subgraph mining process. We performed the search for every vertex of the sample until every possible connection of size 6 was found. Since we did not implement any control during the algorithm, every pattern was likely to be found more than one time and had to be reduced in the following step. This method allowed us to parallelize the search in several machines to reduce computational time.

#### Generate the graph

Next, we generated graphs for each sample, considering the cluster centers as the vertices, and the unique edges as the connecting edges of the vertices.

#### Subgraph mining

The used method for subgraph mining visited the graph through depth-first search, allowing parallelism, e.g. by splitting each starting vertex to be processed at the same time. At every vertex, we looked for all the possible connected paths of size 1. Then, these subgraphs were the candidates for looking for all the possible connected paths of size 2. The process was repeated for the paths of sizes 3, 4, 5, and 6. A graphic representation of this process can be found in Fig. 4 and the corresponding pseudocode in Algorithm 2.

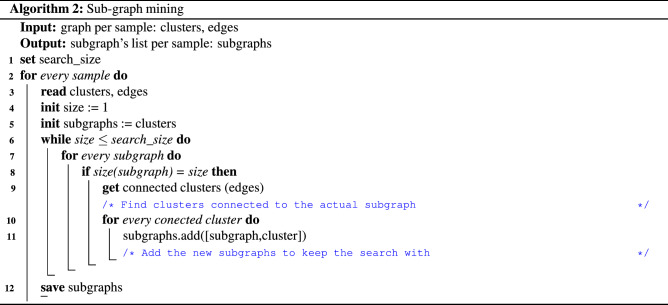



#### Reduce similar patterns

All of the subgraphs obtained from the vertices from a given sample were stored together and duplicated cases were eliminated by matching canonical labels and edge hashes.

### Defining statistically significant patterns

In order to discern statistically significant patterns from random distributions, we compared frequencies between real observations and random observations from simulated datasets using a measure called Abundance ($\Delta$), proposed by Wong^11^.

#### Abundance measure

As defined in (1), we computed $\Delta$ for a given *cycle*, comparing $f_{input}$, which is defined as the frequency of a pattern in the original dataset with $\overline{f}_{random}$, the mean of the frequencies of a pattern in N simulated random datasets. $\varepsilon$ is a pseudo-count (Laplace smoothing) to prevent the ratio from exploding when frequencies are small. $\Delta$ can take values between -1, underrepresented and +1, overrepresented, being 0 the value for a pattern with the same representation in the original data than in the random datasets.

1 $$\begin{aligned} \Delta = \frac{f_{input}-\overline{f}_{random}}{f_{input} + \overline{f}_{random} + \varepsilon } \end{aligned}$$

#### Dataset simulation test

In order to keep the same distribution of clusters as the original dataset, we randomized the edges between the clusters (the rearrangements). The randomization of the edges was performed using an adaptation of the switching method presented by Wong^11^ to the graph abstraction previously described above. This method consists of repeatedly selecting two random edges A–B and C–D and exchanging the ends to form two new edges, e.g, A–D and B–C. The resulting graph keeps the same vertices and edges count. This method has a drawback: we cannot be certain when the graph is adequately randomized, but numerical studies have shown that enough random switching samples (100 $\times$ E) are adequate to achieve a randomized set, where E is the total number of edges across all samples^21^. Therefore, we generated 100 simulated datasets as follows: we removed the original edges of every sample and randomly assigned the same amount of edges to each sample every time.

## Results

### Clusters of SVs from complex patterns

The purpose of the clustering process is to join the rearrangements that belong to the same mutation event. Therefore, in order to select the optimal bandwidths and carry out the 2-step KDE clustering, we ran several experiments with different bandwidth values, observing that the resolution of a 1-step KDE clustering is limited by the size of the chromosomes; the density estimation was exactly the same using any bandwidth equal or smaller than 1000. A first inspection of the results showed low resolution, as breakpoints were being clustered despite being separated by hundreds of thousands base pairs, indicating the need to perform a second clustering to improve the resolution since two SVs can not be considered the same event being that far apart^5^. This is happening because hundreds of thousand base pairs is considered a small distance when applying the method to a whole chromosome that contains between 50 and 250 million base pairs.

**Figure 5:**
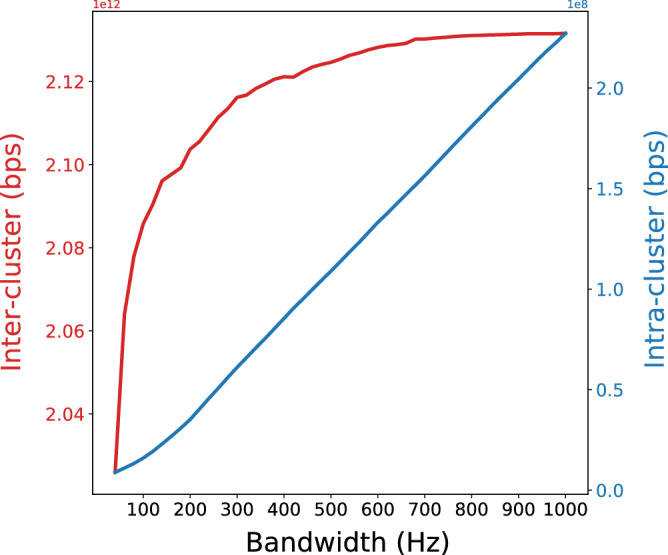
Total inter and intra-cluster distances for the whole dataset using the 2-step KDE clustering with different bandwidth values.

The final selected values for the method were bandwidth 1 = 1000 for the first step since it ensured the maximum resolution and bandwidth 2 = 400 for the second step since it showed high inter-cluster distances while still having small intra-cluster distances. As seen in Fig. 5, selecting a higher bandwidth the breakpoints were clustered with a considerable increase of the intra-cluster distance while almost not increasing the inter-cluster distance. Opposite, selecting a lower bandwidth the behavior was smaller intra-cluster distance but with a significant decrease in the inter-cluster distance.

To determine whether the obtained clusters were composed by random rearrangements, we first analyzed the distribution of the breakpoints in the original dataset. After comparing the dispersion of breakpoints in the simulated datasets with the dispersion from the original dataset, we got a *p*-value smaller than $1^{-5}$, indicating that the breakpoint locations were not following a random distribution in the cancer genomes. Furthermore, we compared the cluster density in the simulated data and the original dataset finding that the cluster density of the original dataset was unlikely obtained in a random simulation (*p*-value $< 1^{-5}$). Therefore, the clusters we obtained implementing the 2-step KDE clustering contain SVs that are likely mechanically linked and not just random occurrences.

### Motif finding

Using the graph mining technique allowed us to to convert our pattern search across all the genome of every sample in a simpler graph search. Within High Performing Computing environments that are based on Apache HBase^22^, HDFS^23^ and Spark^24^ we are able to distribute the computational load across several machines. We used three machines with an Intel$\circledR$ Xeon(R) CPU E5-2630 v4 @2.20GHz processor, 128MB of RAM, and 20 cores each. Using these technologies, the search across 2392 samples was done in less than a day. The use of High Performing Computing methods becomes crucial for the analysis of simulated datasets, where we must repeat the methodology for 100 simulations.

Here, we only focused on *cycles* limited to a size of 6. The *cycle* with a size of 3, named *triangle*, was the pattern more recurrent across the different cancer samples. Its confidence was almost twice the confidence of the next simplest *cycle*, composed of only 1 edge more (Table 1).

**Table 1:** Statistical values for the evaluated *cycles*. The values obtained are defined as follows. Confidence, which provides the number of samples that have at least one *cycle* occurrence. Average which refers to the average of the number of *cycles* happening in the samples. And finally, frequency, the sum of all the occurrences of the *cycle* across the whole dataset.

Cycle size	Confidence	Average	Frequency
3	814	4.68	3817
4	417	6.75	2817
5	260	4.04	1051
6	188	44.43	8354

**Figure 6:**
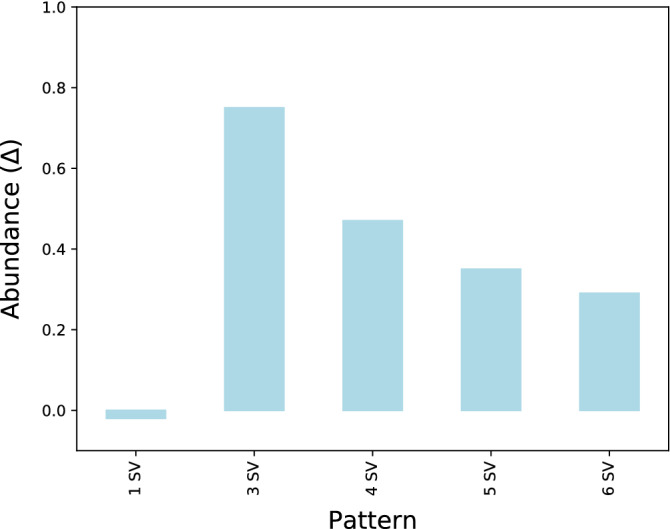
Abundance values for the analyzed *cycles*. Its value can go from − 1, underrepresented, to + 1, overrepresented. The Abundance of a single rearrangement (1 SV) is also shown as a control value. Its value is 0 since we fix the rearrangements during the simulation of the random datasets, which means that its representation is the same in every dataset.

The challenge in the identification of complex patterns is to discern between the distributions of rearrangements that are the sum of random unrelated occurrences from those that are mechanically associated. We measured the significance of the patterns by calculating the Abundance ($\Delta$). All the *cycles* evaluated in this study were overrepresented as shown in Fig. 6 (all *cycles* got positive values of Abundance.) However, as the number of rearrangements of the *cycle* increased, the Abundance decreased, being the *triangle*, the most overrepresented pattern.

### Pattern significance across cancer types

Analyzing the behavior of the *cycles* in each cancer type, the abundances differed between tumor types (see Fig. S2). The *triangle* pattern again predominated over the majority of cancers, with the exceptions of Bone-Osteosarc, Kidney-ChRCC, Lymph-CLL, and Uterus-AdenoCA. Furthermore, there are tumor types that were more similar in terms of abundances of particular *cycles*. For example, Bladder-TCC, Bone-Osteosarc, Breast-AdenoCA, Breast-LobularCA, ColoRect-AdenoCA, Eso-AdenoCA, Head-SCC, Kidney-ChRCC, Lung-AdenoCA, Lung-SCC, Ovary-AdenoCA, Panc-AdenoCA, Prost-AdenoCA, SoftTissue-Leimyo, Stomach-AdenoCA, Uterus-AdenoCA had high Abundance for most of the *cycles*. In contrast, Breast-DCIS, Cervix-AdenoCA, Myeloid-AML, Myeloid-MPN had Abundance = 0 for every *cycle* or almost every *cycle*. This group was clearly composed of cancer types without enough samples or complexity. The rest of the cancer types lied somewhere in the middle, having Abundance values not as high as the first group but not having all of them to 0 either: Biliary-AdenoCA, Bone-Benighm, Bone-Epith, CNS-GBM, CNS-Medullo, CNS-Oligo, CNS-PiloAstro, Cervix-SCC, Kidney-RCC, Liver-HCC, Lymph-BNHL, Lymph-CLL, Panc-Endocrine, Skin-Melanoma, SoftTissue-Liposarc, Thy-AdenoCA.

### Characterization of triangle types

We further characterized the *triangle* pattern since it was the most overrepresented and recurrent across all the samples. Known patterns of structural variants that could coincide with these *triangles* have been described based on the orientation of chromosomal segments at the breakpoints and their associated copy-number alterations. Using these criteria, we subclassified the *triangle* patterns into four different categories: (i) Chromoplexy described by Baca et al.^3^ where usually there is not DNA gain and even, there could be a minimal loss (balanced rearrangements); (ii) Cycles of templated insertions, characterized by copy number gains and inverted rearrangements^5^; (iii) Non-canonical chromothripsis, a pattern that was recently described^25^, which can involve different chromosomes with frequently inverted rearrangements with oscillating copy-number alterations; (iv) The fourth pattern, that we here have called Chromotrikona (from the Greek chromo for chromosome and from the Sanskrit trikona for triangle), do not correspond to any other pattern previously described and is characterized by the presence of frequent inverted rearrangements with no significant gains or losses of DNA.

Once we set the four classes of *triangles*, their abundances were estimated (see Fig. S3). Since we already knew that *triangles* were overrepresented, we expected to have a high abundance in all types. However, we noticed that Chromoplexy and Chromotrikona patterns were the most overrepresented types. These abundance similarities may be generated due to an overlapping of *triangles* of both types, having one or more clusters in common. Since we knew that clusters could have more than one breakpoint, they could be linked to different clusters, forming different *triangles* and therefore, different *triangle* types. We calculated the number of clusters that had in common every pair of *triangles* (see Fig. S4). As expected, Chromoplexy patterns had more common clusters with Chromotrikona patterns. Furthermore, this behavior was also maintained for Cycles of templated insertions and Non-canonical chromothripsis. These results suggest that these patterns could share some underlying properties as they are found in the same genomic regions.

**Figure 7:**
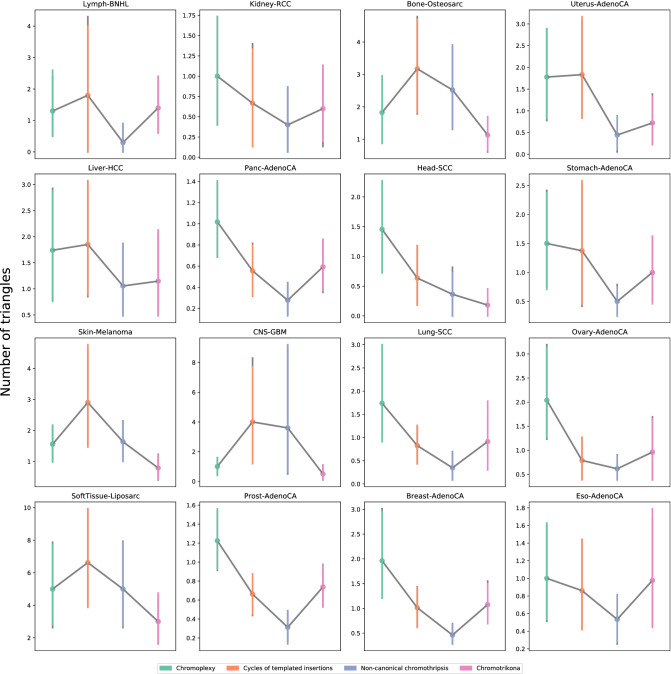
Confidence intervals of the mean of the frequency for each *triangle* type throughout cancer types. Only cancers with more than 10 samples with *triangles* were showed.

We also performed an analysis of how these *triangle* types were distributed among the different cancer types. We excluded cancer types having less than 10 samples with *triangles* to avoid possible bias due to the low number of samples. The presence of the *triangle* types were heterogeneous across cancer types (Fig. 7). For instance, Chromoplexy was more common than the other *triangle* types in Kidney-RCC, Uterus-AdenoCA, Panc-AdenoCA, Head-SCC, Ovary-AdenoCA, Prost-AdenoCA, and Breast-AdenoCA, while Cycles of Templated Insertions was predominant in Bone-Osteosarc or Skin-Melanoma. Chromotrikona predominated only in Kidney-RCC and was the less represented pattern in Bone-Osteosarc, Liver-HCC, Head-SCC, Skin-Melanoma and SoftTissue-Liposarc.

## Discussion

The identification and classification of complex patterns in cancer genomes are not well explored. The complexity of the data and the lack of certainty about the relevant cases claims new strategies that allow us to get insights into their underlying role in tumorigenesis.

Here we have proposed a statistical framework to fulfill this gap. First, we used a KDE-based clustering method identifying adjacent SVs that are not independent events but belonged to the same single event. The KDE clustering has been proven to be fast and simple and very suitable for distribution based-clustering tasks without setting a priori number of clusters^26,27^. Facing the lack of reference complex patterns of SVs to compare with, we presented a statistical approximation to prove that the clusters of SVs were not by chance, indicating that they must be related to each other^28,29^.

For the detection of motifs to identify the complex chromosomal rearrangements, we adapted a graph mining strategy with a measure of significance for each found pattern^30^. Similar motif finding algorithms based on randomizations have been already proved successfully such as FANMODE^31^, MODA^32^, and NetMode^33^. All these studies agree that the need to apply the methods to both the original and simulated datasets translates into a high computational burden. We used parallelization and HPC tools to decrease the computational cost of the method^34^, as well as narrow down the search to patterns of size 6. The selected measure for analyzing the significance of the motifs, the Abundance, is directly related to the *z*-score of the pattern but normalized, allowing us to compare among different patterns^35^.

Taken together, we here present the development and application of a new methodology for the classification of complex SV patterns in tumor genomes. Applying this method to more than 150 thousand SVs from the PCAWG cohort we could identify existing known patterns, as well as a new pattern (Chromotrikona) composed of three SVs that involves balanced inversions between distinct DNA regions in 2 or 3 chromosomes. This represents a significant step forward towards the understanding of the role of complex structural rearrangements in cancer.

## Conclusions

In this study, we presented the development of a new statistical strategy for the classification of complex rearrangements in cancer, which is key to understanding the role and the impact of structural variation in the origin and evolution of tumors. Considering the current expansion of AI approaches for the analysis of complex biological data, this study highlights the necessity to establish robust, unbiased, and accurate statistical frames that are the foundation of more complex machine learning algorithms.

The new strategy proposed in this study fulfilled this end, being composed of a novel application of a clustering solution based on the data distribution, a robust motif finding algorithm that can be easily parallelizable to decrease the computational cost of such an extensive search and a final statistical measure that accurately ranks the obtained patterns in terms of significance.

The results showed the identification of different known patterns in cancer samples as well as a new pattern not previously described. This recurrent pattern, called Chromotrikona, is defined by inverted rearrangements where there are no significant gains or losses of DNA. The development of methods for studying complex patterns of SVs allows us to have insights into new patterns but also understand the genesis of chromosomal rearrangements without limited resolutions. Such genomic rearrangements are the result of subverted biological processes by which they contribute to cancer development.

## Supplementary Information

Supplementary Information.

## Data Availability

All the data analyzed during the current study are available in the data repositories from ICGC data portal.
