# Author Correction: Clustering and graph mining techniques for classification of complex structural variations in cancer genomes

**DOI:** 10.1038/s41598-022-09758-w

**Published:** 2022-04-04

**Authors:** Gonzalo Gomez-Sanchez, Luisa Delgado-Serrano, David Carrera, David Torrents, Josep Ll. Berral

**Affiliations:** 1 Department of Computer Science, Barcelona Supercomputing Center (BSC), 08034 Barcelona, Spain; 2 Universitat Politècnica de Catalunya (UPC), 08034 Barcelona, Spain; 3 Department of Life Science, Barcelona Supercomputing Center (BSC), 08034 Barcelona, Spain; 4 Institució Catalana de Recerca i Estudis Avançats (ICREA), 08010 Barcelona, Spain

Correction to: *Scientific Reports* 10.1038/s41598-022-07211-6, published online 28 March 2022

The Acknowledgements section in the original version of this Article was incomplete.

“We acknowledge the access to data from PCAWG Consortium which provided SVs data. We thank the patients and their families for their participation in the ICGC and TCGA projects. Among others, this study has been supported by projects: SAF2017-89450-R (TransTumVar) and PID2020-119797RB-100 (BenchSV) from Science and Innovation Spanish Ministry. It has also been supported by the Spanish Government (contract PID2019-107255GB), Generalitat de Catalunya (contract 2014-SGR-1051) and Universitat Politècnica de Catalunya (45-FPIUPC2018).”

now reads:

“We acknowledge the access to data from PCAWG Consortium which provided SVs data. We thank the patients and their families for their participation in the ICGC and TCGA projects. Among others, this study has been supported by projects: BenchSV (PID2020-119797RB-I00/AEI/10.13039/501100011033) from Science and Innovation Spanish Ministry and SAF2017-89450-R (TransTumVar). It has also been supported by the Spanish Government (PID2019-107255GB-C21/AEI/10.13039/501100011033), Generalitat de Catalunya (contract 2014-SGR-1051) and Universitat Politècnica de Catalunya (45-FPIUPC2018).”

The original Article has been corrected.

