# Supplementary material for: Clustering and graph mining techniques for classification of complex structural variations in cancer genomes
Source: Sci Rep. 2022 Feb 28;12:3244. doi: 10.1038/s41598-022-07211-6 (PMC8885672; doi:10.1038/s41598-022-07211-6)
Supplement: Supplementary file 1 — Supplementary Information. [file 41598_2022_7211_MOESM1_ESM.pdf]

Cancer Type	Number of Samples
Biliary-AdenoCA	31
Bladder-TCC	23
Bone-Benign	9
Bone-Epith	9
Bone-Osteosarc	37
Breast-AdenoCA	186
Breast-DCIS	3
Breast-LobularCA	12
CNS-GBM	40
CNS-Medullo	102
CNS-Oligo	17
CNS-PiloAstro	11
Cervix-AdenoCA	2
Cervix-SCC	18
ColoRect-AdenoCA	60
Eso-AdenoCA	87
Head-SCC	55
Kidney-ChRCC	32
Kidney-RCC	114
Liver-HCC	325
Lung-AdenoCA	35
Lung-SCC	47
Lymph-BNHL	105
Lymph-CLL	77
Myeloid-AML	7
Myeloid-MPN	4
Ovary-AdenoCA	112
Panc-AdenoCA	237
Panc-Endocrine	55
Prost-AdenoCA	270
Skin-Melanoma	106
SoftTissue-Leiomyo	15
SoftTissue-Liposarc	17
Stomach-AdenoCA	70
Thy-AdenoCA	19
Uterus-AdenoCA	43

Table 1: Number of tumor genome samples for each cancer type used from PCAWG dataset. Samples with less than 3 SVs have not been selected, since they can not form complex rearrangements (not included in the table).

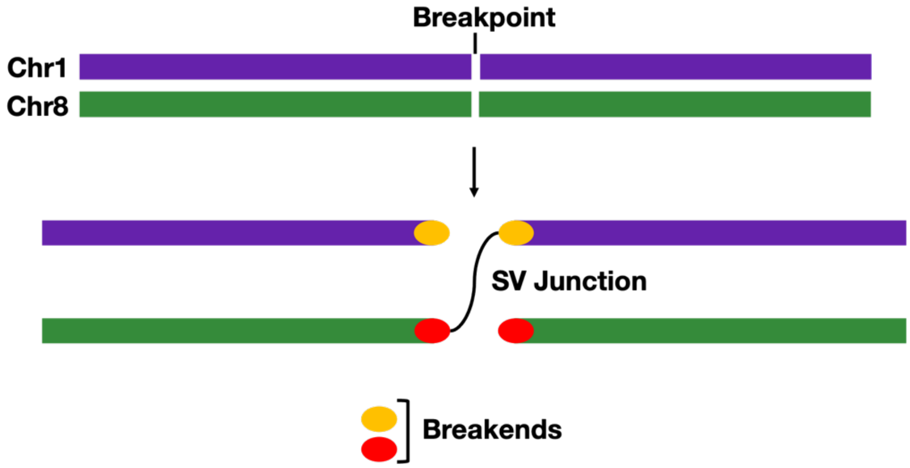

Figure S1: Schematic representation of breakpoints, breakends, and SV junctions. In this example, chromosome 1 (Chr1) and chromosome 8 (Chr8) are broken at two genomic positions or breakpoints, generating two breakends per chromosome, shown in yellow and red spots, respectively. The SV, in this case, an interchromosomal translocation, results from the cross-joining of the backends. The black line shows the SV junction.

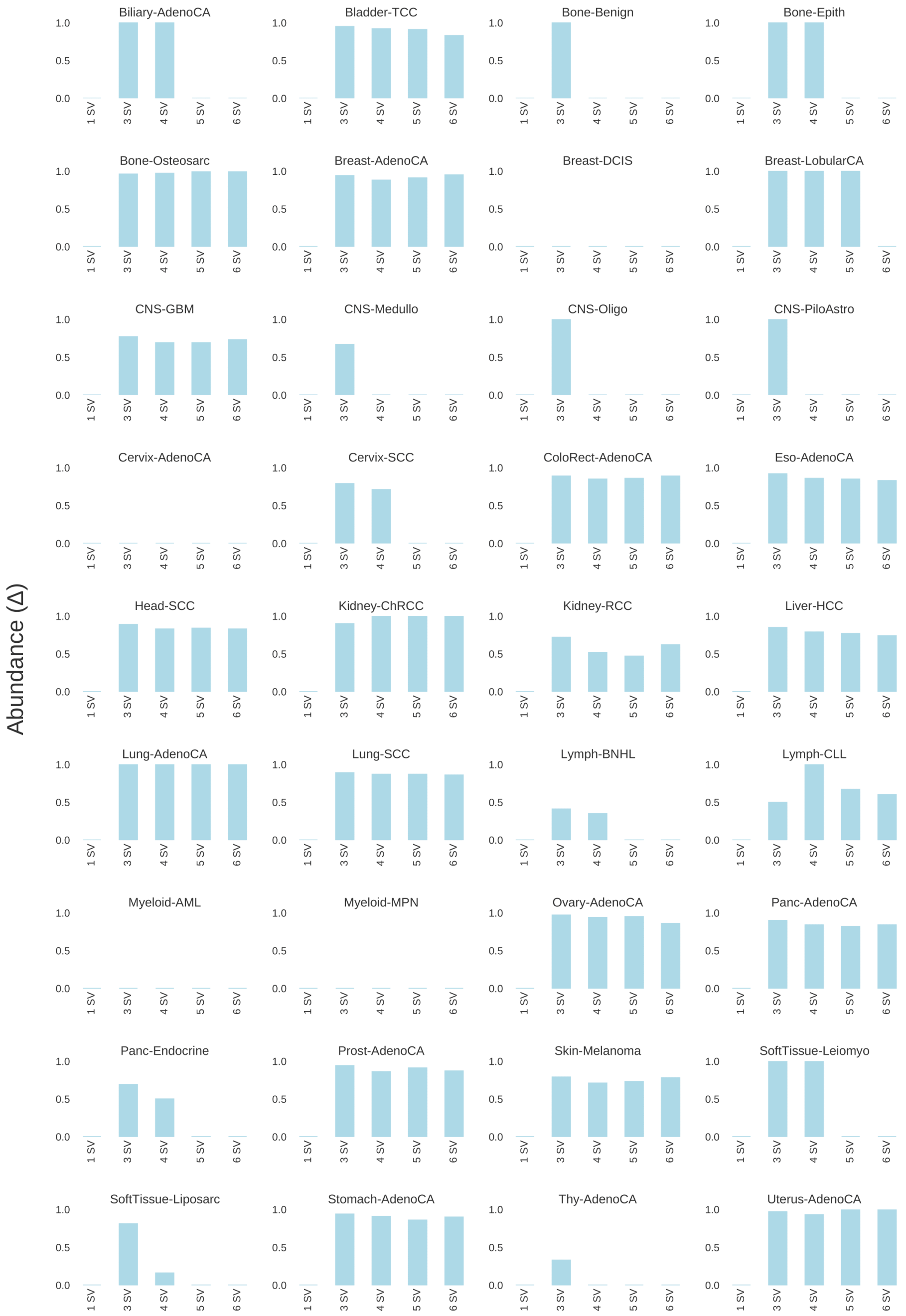

Figure S2: Abundance values of the evaluated cycles for the 36 cancer types. Cycles with an Abundance = 0 do not have any pattern both in the simulation and in the original dataset.

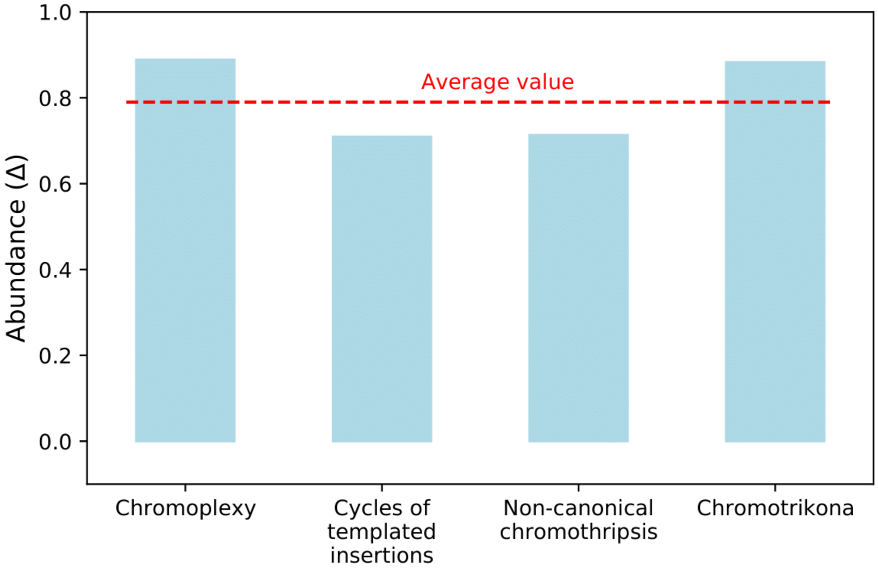

Figure S3: Abundance values for different triangle categories.

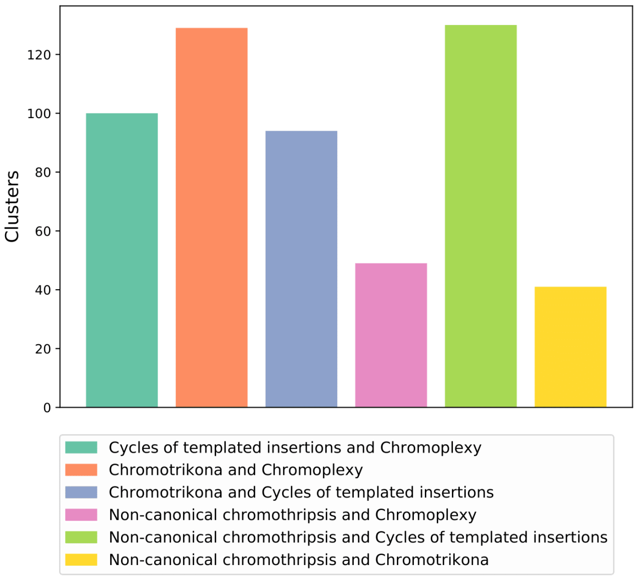

Figure S4: Common clusters between every pair of triangle types.
